# Socioeconomic Risk Factors of Poor Nutritional Status in Polish Elderly Population: The Results of PolSenior2 Study

**DOI:** 10.3390/nu13124388

**Published:** 2021-12-08

**Authors:** Roma Krzymińska-Siemaszko, Ewa Deskur-Śmielecka, Aleksandra Kaluźniak-Szymanowska, Beata Kaczmarek, Hanna Kujawska-Danecka, Alicja Klich-Rączka, Małgorzata Mossakowska, Sylwia Małgorzewicz, Lechosław B. Dworak, Tomasz Kostka, Jerzy Chudek, Katarzyna Wieczorowska-Tobis

**Affiliations:** 1 Department of Palliative Medicine, Poznan University of Medical Sciences, 61-245 Poznan, Poland; edeskur@ump.edu.pl (E.D.-Ś.); olakaluzniak@gmail.com (A.K.-S.); be.kaczmarek@ump.edu.pl (B.K.); kwt@tobis.pl (K.W.-T.); 2 Clinic of Internal Medicine, Connective Tissue Diseases and Geriatrics, Medical University of Gdansk, 80-210 Gdansk, Poland; hanna.kujawska@gumed.edu.pl; 3 Department of Internal Medicine and Gerontology, Collegium Medicum of Jagiellonian University, 31-008 Krakow, Poland; ala_klich@o2.pl; 4 International Institute of Molecular and Cell Biology, 02-109 Warsaw, Poland; mmossakowska@iimcb.gov.pl; 5 Department of Clinical Nutrition, Medical University of Gdansk, 80-210 Gdansk, Poland; sylwia.malgorzewicz@gumed.edu.pl; 6 Faculty of Health Sciences, Calisia University, 62-800 Kalisz, Poland; biomechanika-dworak@wp.pl; 7 Healthy Ageing Research Centre (HARC), Department of Geriatrics, Medical University of Lodz, 90-647 Lodz, Poland; tomasz.kostka@umed.lodz.pl; 8 Department of Internal Medicine and Oncological Chemotherapy, Medical University of Silesia, 40-055 Katowice, Poland; chj@poczta.fm

**Keywords:** poor nutritional status, malnutrition, older adults

## Abstract

Poor nutritional status (PNS) threatens successful aging. Identifying potentially modifiable predictors of PNS is essential for elaborating a preventive strategy for the population at risk. To assess the prevalence of PNS in the Polish elderly population and analyze its socioeconomic correlates based on the data from the nationwide PolSenior2 project. Special emphasis was put on potentially modifiable factors among the identified PNS predictors. Nutritional status was assessed in 5698 community-dwelling older adults with the Mini Nutritional Assessment–Short Form. We evaluated the effect of age, sex, level of education, marital status, place of residence, subjective loneliness, and self-reported poverty on the nutritional status of the studied subjects. PNS was found in 25.3% of studied subjects (27.7% women and 21.9% men; *p* < 0.001). Female sex, older age, unmarried status (in men), subjective loneliness, and self-reported poverty were independent correlates of PNS. The two last above-mentioned predictors were identified as potentially modifiable. Based on our results, we recommend preventive interventions (e.g., performing regular screening), particularly in unmarried (men), poorly educated individuals, self-reporting poverty, complaining of loneliness, and the oldest old. PNS preventive strategies should include social support (both emotional and instrumental) to reduce the effect of poverty and subjective loneliness.

## 1. Introduction

Aging is associated with the accumulation of disorders that may negatively affect the balance between nutritional intake and body requirements [1]. Adequate nutrition is crucial in successful aging, preventing diet-dependent diseases, and preserving good health [2]. Some form of malnutrition (quantitative and/or qualitative undernutrition) affects many elderly subjects living both in developed and developing countries, resulting in decreased functional capacity [3]. Poor nutritional status impairs quality of life and increases the risk of institutionalization, morbidity, and mortality in older adults [4,5,6]. It should be emphasized that malnutrition is a potentially modifiable condition. Like other health problems in the older population, it is easier to prevent than treat [7].

Appropriate screening for malnutrition enables the identification of subjects at risk, who consequently should be offered nutritional support. According to the European Society for Clinical Nutrition and Metabolism (ESPEN), the Mini Nutritional Assessment questionnaire (MNA) is the most frequently used nutritional assessment tool among older subjects [8,9]. The unquestionable advantage of MNA is that it refers to the subject’s physical, psychological, and cognitive status, thus assessing multiple risk factors for malnutrition [9]. Unfortunately, the diagnosis of malnutrition is frequently neglected in clinical practice, and the condition remains frequently underdiagnosed worldwide [5,10]. Several barriers to screening and successful management of malnutrition have been identified [11].

Many factors contribute to the risk of malnutrition in older subjects, clearly shown in the Determinants of Malnutrition in Aged Persons (DoMap) model. The model comprises 18 direct and 27 indirect malnutrition risk factors associated with three principal conditions involved in the development of malnutrition: low intake, high requirements, and impaired nutrient bioavailability [12]. Indirect factors, such as poverty, may lead to malnutrition by activating a direct factor for one of these three main mechanisms. For example, poverty (indirect factor) may result in a lack of food (a direct factor) and low intake (a mechanism). Thus, some socioeconomic variables may be among the predictive factors for malnutrition.

A recent systematic review (comprising 40 studies) with a meta-analysis of 16 observational studies assessing the risk of malnutrition in various elderly populations confirmed a strong relation between several socioeconomic factors and malnutrition or risk of malnutrition in subjects over 60 [9]. However, most studies on malnutrition in elderly subjects were conducted in residents of nursing homes or health facilities (such as hospitals or ambulatories) and data on older adults living in a community are missing [13]. Population projections in most countries show a distinct increase in the percentage of elderly subjects, most of whom will presumably live in their homes [14]. Therefore, studies investigating the nutritional risk in elderly community-dwellers are crucial to meet future nutritional requirements of this population and develop home-based dietary interventions. As pointed out by O’Keeffe et al. [10], it is essential not only to recognize risk factors for malnutrition but also to verify which of them are modifiable. So far, data on the topic are scarce.

Our analysis aimed to assess the epidemiology of poor nutritional status (PNS) and analyze its social and economic correlates in community-dwelling elderly subjects involved in the PolSenior2 study. Additionally, we intended to identify potentially modifiable PNS predictors among seven analyzed socioeconomic variables.

## 2. Materials and Methods

The PolSenior2 study was conducted in 2018 and 2019 to characterize the medical, psychological, social, and economic status of a large group of old and very old adults in Poland. It involved 5987 participants aged 60 years or more, a representative sample of the elderly Polish population, drawn in equal size 5-year age (60–65, 65–69, 70–74, 75–79, 80–84, 85–89, and 90+ years) sex strata by multistage clustered sampling method with intended overrepresentation of the oldest subjects compared to the contemporary structure of Polish society. The study protocol assumed three visits of a trained 507 interviewer, which took place at the respondent’s home. The interviewers were professionally active nurses who worked mainly in local communities, trained for the purpose of the PolSenior2 project. Nurses attempted to reach 18,695 randomly selected respondents. Out of the 10,635 available respondents, 5987 people were tested (4648 refused). During the first visit, standardized scales for comprehensive geriatric assessment, including Geriatric Depression Scale (GDS), Mini-Mental State Examination (MMSE), and Mini Nutritional Assessment-Short Form (MNA-SF), were applied. The questions were asked directly to the respondent by the interviewer. If the subject could not answer it, it could have been done by a family member or caregiver, and that this fact was noted. The study protocol was approved by the Bioethics Committee of the Medical University of Gdansk (NKBBN/257/2017). Each respondent or his/her caregiver gave informed consent before the study [15]. The analysis of PNS included data of 5698 subjects (2913 women and 2785 men). Data of 289 participants (4.8% of PolSenior2 population) with an incomplete nutritional assessment with the MNA-SF questionnaire were excluded from further analysis.

### 2.1. Nutritional Status

The nutritional risk was assessed with the Mini Nutritional Assessment-Short Form (MNA-SF), a questionnaire developed to screen the nutritional status of persons over 65 years of age as a component of the Comprehensive Geriatric Assessment [16]. The questionnaire contains six items, investigating the decline in food intake and weight loss in the past three months, acute disease or emotional distress in the past three months, mobility, and neuropsychological problems, such as depression or dementia. The last item assesses body mass index [BMI; a ratio of weight (in kilograms) to the square of height (in meters^2^)]; if data necessary to calculate the BMI are missing, calf circumference may be used interchangeably. We used such an option in 111 participants. The maximum MNA-SF score is 14 points. A score of 0–7 points indicates malnutrition, 8–11 points suggest a person is at risk of malnutrition, and a score of at least 12 points shows the normal nutritional status [16]. We pooled all participants with fewer than 12 points in one category–poor nutritional status (PNS). It should be emphasized that the MNA-SF scale has been validated in community-dwelling elderly in Poland by Kostka et al. [17].

Before completing the MNA-SF item concerning neuropsychological problems, we assessed all participants for depression and cognitive impairment. Cognitive status was assessed with the Mini-Mental State Examination (MMSE) corrected by age and education according to Mungas [18]. As currently recommended, a score of 27.0–30.0 points was regarded as normal. Abnormal results, indicating possible cognitive impairment, were classed as mild cognitive impairment (24.0–26.99 points), mild dementia (19.0–23.99 points), moderate dementia (11–18.99 points), and severe dementia (0–10.99 points) [19].

We assessed the risk of depression with the short version of the Geriatric Depression Scale (GDS), composed of 15 questions [20]. The GDS is assumed to be self-completed by the respondents. However, in the PolSenior2 project, nurses filled in the questionnaire based on participants’ answers to verbal questions. Such an attempt enabled obtaining data from participants with a very low level of education and subjects with vision problems (much more frequent than severe hearing problems). Geriatric Depression Scale was omitted in participants with the MMSE score of fewer than 19 points to rule out the unreliable answers given by subjects suspected of moderate/severe dementia. Subjects who received at least 6 points in the GDS were classified as having depression symptoms [20].

### 2.2. Data Collection on Socioeconomic Characteristics

In addition to the MNA-SF questions, we collected data on participants’ sex, age, marital status, place of living, level of education, subjective loneliness, and self-reported poverty during a home interview conducted by nurses deliberately trained for the study aim and specificity. In the PolSenior2 project participants’ financial condition was assessed using six variants of answers: (1) financial resources not sufficient to buy even cheapest food and clothes, (2) financial resources sufficient to buy only cheapest food and clothes, (3) financial resources sufficient to buy only cheapest food, not sufficient to buy clothes, (4) sufficient financial resources, no need for money-saving, (5) I am/we are saving money for some extra expenditures, (6) I am/we are economizing and I/we have enough resources for living. Respondents who had chosen answer variants 1, 2, or 3 were classified in the self-reported poverty group. We assessed subjective loneliness with a question: ‘How often do you feel lonely?’ and the following answer variants: ‘never’, ‘rarely’, ‘sometimes’, ‘often’, ‘always’, and ‘it is difficult to say’. Respondents who had chosen ‘never’, ‘rarely’, ‘sometimes’, and ‘it is hard to say’ were pooled into one category ‘without subjective loneliness’.

### 2.3. Statistical Analysis

We performed statistical analysis and presentation of data with the R statistical package (R Core Team, version 3.6.3.). We included sampling weights in calculations of proportions (prevalence rates) and 95% confidence intervals to account for the complex survey design. The post-stratification procedure was used to match the age-sex sample distribution to the national population and calculate the population prevalence of PNS and malnutrition. We assessed the relationship between PNS and a set of analyzed risk factors with unweighted logistic regression. A step-wise backward selection procedure was used to build a multivariable model. Regression coefficients were presented as odds ratios with a 95% confidence interval. We used the unpaired t-test and the chi-square test to assess the between-group differences for quantitative and categorical data, respectively. The 2-tailed tests were performed at a significance level of *p* < 0.05.

## 3. Results

### 3.1. Study Participants Characteristics

The analyzed population consisted of 5698 participants aged 60–106 years (mean age 74.8 ± 9.4 years), 51.1% were women (*n* = 2913). Nearly 40% of participants were unmarried (1586 women and 602 men), including widows/widowers (*n* = 1805; 1356 women), those who were divorced or separated from their spouses (*n* = 217; 135 women), as well as those never married (*n* = 166; 95 women). At most elementary education (elementary education, incomplete elementary education, or no formal education) was declared by 28.8% of the individuals (*n* = 1607; 949 women). Most respondents (64.9%; 1877 women) lived in urban areas. Slightly above seven percent of all studied subjects (*n* = 401; 246 women) declared that they could afford to buy only the cheapest food/clothing or not even that, which was considered as self-reported poverty. A similar number of participants (*n* = 429; 278 women) stated that they often or always experience loneliness.

### 3.2. Factors Associated with Poor Nutritional Status

**Figure 1:**
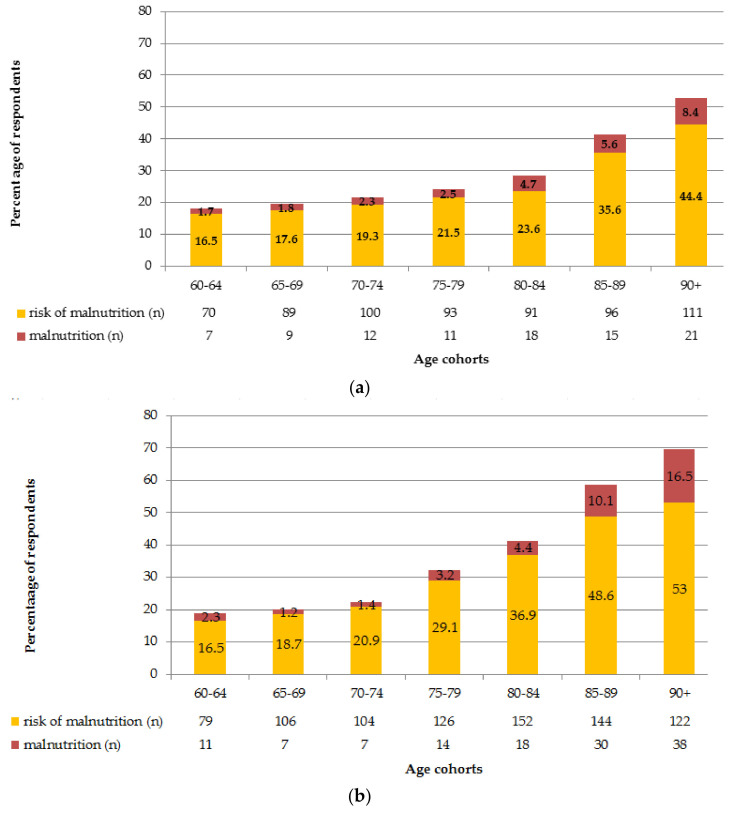
(**a**). The frequency of malnutrition and its risk in the Polish older men stratified by age. (**b**). The frequency of malnutrition and its risk in the Polish older women stratified by age.

The prevalence of PNS and malnutrition in Polish older adult population was estimated at 25.3% (27.7% women and 21.9% men; *p* < 0.001) and 2.8% (3.1% women and 2.3% men; *p* = 0.07), respectively. The frequency of malnutrition and is risk-stratified for age, separately for men and women is presented in Figure 1a,b.

Table 1 shows the impact of social and economic parameters on the PNS and normal nutritional status frequency in univariable analysis. PNS was observed 1.2 times more frequently in women than in men (32.9% vs. 26.7%; *p* < 0.001). Age had a strong influence on the frequency of PNS. Poor nutritional status was diagnosed over 3 times more frequently in the oldest subgroup (90+) as compared to the youngest one (age 60–65; 60.8% vs. 18.5%; *p* < 0.001). Married individuals had PNS two times less frequently than single ones (40.2% vs. 22.9%; *p* < 0.001). Subjects with the lowest level of education (elementary, incomplete elementary, or no formal education) had PNS two times more frequently as compared to individuals with a higher level of education (43.6% vs. 22.6%; *p* < 0.01). Poor nutritional status was more frequent in respondents living in rural areas (32.1% vs. 28.6%; *p* < 0.001) and in subjects declaring self-reported poverty (43.4% vs. 28.2%; *p* < 0.001). Individuals always or often experiencing loneliness had a 2.3 times higher risk of PNS (60.6% vs. 26.8%; *p* < 0.001).

### 3.3. Multivariable Analysis Results

We included all factors increasing the risk of PNS in univariable analysis in the multiple regression model (sex, age, level of education, place of residence, marital status, self-reported poverty, and subjective loneliness).

The multiple regression models, overall and separately for men and women, are presented in Table 2. Subjective loneliness was the strongest independent correlate of PNS in the population studied. We also found female sex, age (for every ten years), unmarried status, low education level, and self-reported poverty to be independent factors contributing to PNS. Inhabiting a rural area was not a PNS risk factor in the multivariable models.

For both sexes, the common correlates included: age (for every ten years), a low education level (including no formal education), subjective loneliness, and self-reported poverty. In men, PNS was also related to unmarried status. Interestingly, being unmarried did not increase the risk of PNS in women.

## 4. Discussion

### 4.1. Epidemiology of PNS

Poor nutritional status is a common and severe health problem in elderly subjects. Understanding its etiology could be the first step to implementing appropriate prevention and treatment and preventing its consequences. Data from studies performed with the full version of the MNA questionnaire indicate that as many as 25% of elderly Europeans living in the community have a PNS, a 2% may suffer from malnutrition [21]. A similar percentage of subjects with low nutritional status was observed in our study, using the short version of the MNA. These data indicate that one out of four elderly Europeans, including Polish people, needs nutritional support to prevent malnutrition and its negative consequences. The scale of the problem is, therefore, immense.

### 4.2. Socioeconomic Predictors of PNS

As malnutrition has essential negative health consequences in elderly subjects, it is a priority to determine risk factors for PNS in this population. That could be the first step to implementing early prevention and treatment. In our analysis, the strongest socioeconomic predictor of PNS in older Polish living in the community was subjective loneliness. The odds ratio was approximately 3 (OR 3.34 in men and 2.71 in women). In line with our findings, Eskelinen et al. [22] found that feeling lonely was an independent predictor of PNS in a randomly selected sample of elderly Finns; however, the odds ratio for this factor was somewhat lower [OR 1.63 (1.09–2.45)]. Another randomized study conducted in a group of 2106 elderly Norwegians yielded similar results, with an odds ratio of 1.61 for loneliness as a predictor for PNS [14]. Eskelinen et al. suggested that subjective loneliness may reduce appetite and food intake via decreased mood, worsening physical fitness, or impairment in cognitive function [22]. Elderly subjects have a greater risk of bereavement (a spouse, siblings, friends), leading to loss of social relationships, isolation at home, fewer opportunities to meet others, and finally, feeling of loneliness [23].

The risk of malnutrition increased with age in our study population, which agrees with previous investigations [1,5,22]. Similar to other studies, the prevalence of PNS was higher in women [5,22,24,25].

Nearly 40% of our study participants were unmarried, with widows and widowers being in the majority (82.5%). Being unmarried was related to a 1.23 higher risk of PNS in multiple regression analysis for the total study population. Interestingly, separately analyzed multiple regression models for gender demonstrated that this factor was significant in men only. Marital status is an essential social factor and is usually included in analyses concerning nutrition and health. A protective role of marriage in maintaining normal nutritional status in elderly subjects was observed [26,27]. The key role has been contributed to wives taking care of their husbands [28,29]. Research studies conducted at the end of the 20th century documented that many older women were brought up to feel totally responsible for preparing all meals for their families [30]. Most older men regarded nutrition as exclusively women’s domain, which resulted in a lack of abilities necessary for preparing meals [31]. Therefore, losing a spouse, particularly a wife, is closely related to dietary deterioration in elderly subjects [14]. Locher et al. [32] noticed that older men are at greater risk of developing poor nutritional health because they had not been habitually engaged in the process of shopping or cooking. Once they are widowers, they often do not know how to do these tasks for themselves [32]. As stated by Vesnaver and Keller [29], widowhood is an imposed status that changes one of the most important social relationships–a marriage. It has been reported that food intake is often poor in widowed persons, which is attributed to the grief experienced in the bereavement period [22,29]. As eating is usually a social activity, a loss of a domestic partner may lead to a loss of interest in taking meals [29].

Low education level (or lack of formal education) was another PNS risk factor in our analysis. In line with our results, it was observed that elderly subjects with very low education levels usually have minimal knowledge about healthy nutrition, and their diet is monotonous and predisposes them to malnutrition [1,5,25,33].

Another independent risk factor for PNS revealed in our study was self-reported poverty. It has been well recognized that low socioeconomic status fosters PNS in elderly subjects and hampers food security [3,34]. Persons under financial stress may experience loss of appetite [35]. Besora-Moreno et al. [9] emphasized that healthy, fresh food was the most expensive, making it unavailable for older people with limited financial resources. This factor may further increase the risk of malnutrition. Moreover, many indigent elderly subjects have to choose between buying prescribed drugs and healthy food, prioritizing the first option.

### 4.3. Modifiable Socioeconomic Risk Factors for PNS

Effective management of PNS in the community-dwelling elderly subject is challenging. Harris et al. (2019) have identified several barriers to screening and treating this condition [11]. Older adults may underestimate the importance of nutrition in maintaining good health or do not understand the role of malnutrition screening procedures. Another problem can be skepticism towards nutritional recommendations or reluctance to reveal dietary habits. Healthcare specialists, in turn, list a lack of resources for malnutrition screening (staff, time, space, money), problems with integration of its screening into practice, lack of care pathways to guide action after the screening, and even lack of time to treat among barriers to successful management of PNS. Therefore, malnutrition prevention should be a more effective strategy than treatment [36].

O’Keeffe et al. [10] published a systematic review of 23 prospective studies, including all settings. They identified 30 potentially modifiable risk factors for malnutrition categorized into seven domains (such as oral, psychosocial, medication and care, health, physical function, lifestyle, and eating). O’Keeffe et al. [10] emphasized that research on malnutrition in elderly subjects should be focused on determining which factors are really modifiable because they could underly interventions to prevent and treat malnutrition. In the present study, five socioeconomic predictors of PNS were not modifiable (sex, age, marital status, education, place of residence). Nevertheless, subjective loneliness and self-reported poverty are potentially modifiable and should be given special attention.

Besora-Moreno et al. [9] proposed several prevention measures that may help reduce the negative influence of loneliness on nutritional status in older adults: socializing during meals and avoiding eating alone; going to the relative’s home; doing group activities, and sharing meals. These behavioral interventions should be combined with emotional support [29]. Elderly subjects confined in their homes should be identified as early as possible and should be included in interventions to increase social engagement. Vitman Schorr et al. [37] in their study concerning loneliness and malnutrition in older people during the COVID-19 pandemic suggested that a promising strategy could be repeated psychological-nutritional intervention provided by phone or internet. An essential component of PNS management should be instrumental social support in performing tasks like shopping, preparing meals, and eating (including company during meals, which enables some control over the amount of food consumed by an older person). If there are no family members to help, older people at risk of developing PNS should be offered institutional support to meet their social needs [29].

Poverty is another potentially modifiable predictor of poor nutritional status. Older subjects with PNS and limited financial resources should receive governmental help, such as free nutritional services or social programs. An example can be the Meals-On-Wheels program, which delivers meals to individuals at home who cannot purchase or prepare their meals. Payable nutritional services, e.g., diet catering, although potentially effective, are unavailable in elderly subjects with financial problems. A recent systematic review of 13 studies assessing the effectiveness of services delivering meals to older subjects’ homes (Meals-On-Wheels among others) or collective nutrition for elderly persons demonstrated that such services increase energy, protein, and micronutrient intake and reduce the risk of malnutrition [38]. Delivering meals have a favorable influence on elderly subjects’ perceived loneliness and social isolation, promotes social ties, and may be associated with some extra benefits, like help in housework offered by the suppliers [39,40]. Besora-Morena et al. [9] suggested some other helpful solutions for indigent older people, such as buying seasonal products (which reduces costs) and making a shopping list to avoid buying unnecessary things.

### 4.4. Study Limitation

There are several limitations to our study. First, we used a single question to assess subjective loneliness: How often do you feel lonely? An approach and methodology of evaluating subjective loneliness vary significantly between studies. For example, Suzana et al. [41] used De Jong Gierveld Loneliness Scale, while Boulos et al. [42] the modified version of this questionnaire. Maseda et al. [24] measured the frequency of feelings of loneliness with the Spanish version of the Older Americans Resources and Services (OARS). However, subjective loneliness was assessed in numerous studies based on one question [22,23,37]. Secondly, our analysis is a cross-sectional study, which precludes assessment of the causal relationship between variables. Third, the fact that the respondents did not self-complete the GDS questionnaire can be perceived as a source of bias.

The strong point of our analysis is a large and representative sample, including a high number of the oldest participants and men. The obtained results fill the gap in the PNS epidemiology in East-Central Europe and add to the limited knowledge about modifiable risk factors for malnutrition [10,12].

## 5. Conclusions

PNS was detected in one out of four Polish residents aged 60 or more based on the MNA-SF. These findings demonstrate poor nutritional status is a common condition. Early identification of community-dwelling elderly subjects with a high risk of PNS and preventive interventions is crucial to reducing malnutrition’s health burden. Any decrease in the prevalence of malnutrition or limitation of its severity would result in the reduction of health-related costs [24]. Based on our results, we recommend preventive interventions (e.g., performing regular screening), particularly in unmarried (men), poorly educated individuals, self-reporting poverty, complaining of loneliness, and the oldest old. Effective prevention of poor nutritional status with simple methods, including social support and nutritional education, will presumably have a huge, positive influence on seniors’ quality of life.

## Figures and Tables

**Table 1:** Frequency of poor nutritional status (malnutrition or risk of malnutrition based on MNA-SF screening) in subgroups by socioeconomic status.

Variable	Poor Nutritional Status % (*n*)	Normal Nutritional Status % (*n*)	*p* *
Sex			
Women	958 (32.9%)	1955 (67.1%)	<0.001
Men	743 (26.7%)	2042 (73.3%)	
Age group			
60–64 years	167 (18.5%)	734 (81.5%)	<0.001
65–69 years	211 (19.7%)	860 (80.3%)	
70–74 years	223 (21.9%)	794 (78.1%)	
75–79 years	244 (28.2%)	621 (71.8%)	
80–84 years	279 (35.0%)	519 (65.0%)	
85–89 years	285 (50.4%)	281 (49.6%)	
90 and over	292 (60.8%)	188 (39.2%)	
Level of education			
Primary or less than primary	700 (43.6%)	907 (56.4%)	<0.001
Higher than primary	958 (24.1%)	3021 (75.9%)	
Marital status			
Unmarried	880 (40.2%)	1308 (59.8%)	<0.001
Married	768 (22.9%)	2591 (77.1%)	
Place of residence			
City	1058 (28.6%)	2640 (71.4%)	<0.01
Rural area	643 (32.1%)	1357 (67.8%)	
Subjective loneliness			
Without subjective loneliness	1364 (26.8%)	3731 (73.2%)	<0.001
Often or always	260 (60.6%)	169 (39.4%)	
Self-reported poverty			
Yes	174 (43.4%)	227 (56.6%)	<0.001
No	1439 (28.2%)	3671 (71.8%)	

Notes: * Chi^2^ square.

**Table 2:** Factors associated with poor nutritional status (multiple logistic regression, backward selection model).

Variable	Overall		Men		Women	
	OR (95% Cl)	*p*	OR (95% Cl)	*p*	OR (95% Cl)	*p*
Sex (women)	1.15 (1.00–1.32)	0.046	-		-	
Age (for every 10 years)	1.69 (1.57–1.82)	<0.001	1.52 (1.37–1.68)	0.001	1.93 (1.72–2.15)	<0.001
Education (primary or less than primary)	1.51 (1.30–1.74)	<0.001	1.42 (1.14–1.77)	0.002	1.54 (1.26–1.88)	<0.001
Place of residence (rural areas)	1.07 (0.93–1.23)	0.35	1.10 (0.90–1.34)	0.35	1.04 (0.86–1.26)	0.70
Unmarried status	1.26 (1.09–1.46)	0.002	1.48 (1.19–1.84)	0.001	1.05 (0.86–1.28)	0.65
Subjective loneliness (often or always)	2.95 (2.36–3.68)	<0.001	3.34 (2.31–4.85)	0.001	2.71 (2.05–3.59)	<0.001
Self-reported poverty	1.47 (1.17–1.84)	0.001	1.49 (1.04–2.15)	0.03	1.45 (1.08–1.95)	0.013

## Data Availability

Reasoned requests for access to the database should be addressed to the Scientific Council of PolSenior2 Project (contact K.W-T.).
